# A Simple Low-Cost Flexible Plasmonic Patch Based on Spiky Gold Nanostars for Ultra-Sensitive SERS Sensing

**DOI:** 10.1039/d3an02246c

**Published:** 2024-03-25

**Authors:** Supriya Atta, Aidan J. Canning, Tuan Vo-Dinh

**Affiliations:** a Fitzpatrick Institute for Photonics, Duke University, Durham, NC 27708, USA.; b Department of Biomedical Engineering, Duke University, Durham, NC 27708, USA.; c Department of Chemistry, Duke University, Durham, NC 27708, USA.

**Keywords:** gold nanostars, sharp-branched, crystal violet, plasmonics, SERS, Raman, flexible sensor

## Abstract

Recently, transparent and flexible surface-enhanced Raman scattering (SERS) substrates have received great interest for direct point-of-care detection of analytes on irregular nonplanar surfaces. In this study, we proposed a simple cost-effective strategy to develop a flexible SERS patch utilizing multibranched sharp spiked gold nanostars (GNS) decorated on a commercially available adhesive Scotch Tape for achieving ultra-high SERS sensitivity. The experimental SERS measurements were correlated with theoretical finite element modeling (FEM), which indicates that the GNS having a 2.5 nm branch tip diameter (GNS-4) exhibits the strongest SERS enhancement. Using rhodamine 6G (R6G) as a model analyte, the SERS performance of the flexible SERS patch exhibited a minimum detection limit of R6G as low as 1 pM. The enhancement factor of the SERS patch with GNS-4 was calculated as 6.2× 10^8^, which indicates that our flexible SERS substrate has the potential to achieve ultra-high sensitivity. The reproducibility was tested with 30 different spots showing a relative standard deviation (RSD) of SERS intensity of about 5.4 %, indicating good reproducibility of the SERS platform. To illustrate the usefulness of the flexible SERS sensor patch, we investigated the detection of a carcinogenic compound crystal violet (CV) on fish scales, which is often used as an effective antifungal agent in the aquaculture industry. The results realized the trace detection of CV with the minimum detection limit as low as 1 pM. We believe that our transparent, and flexible SERS patch based on GNS-4 has potential as a versatile, low-cost platform for real-world SERS sensing applications on nonplanar surfaces.

## INTRODUCTION

Surface-enhanced Raman scattering (SERS) has been considered an excellent analytical technique that can provide unique spectral fingerprint information corresponding to the specific structural vibration of the target molecules and has been widely used across various fields with numerous applications such as food safety, biomedical sensing, early disease detection, environmental surveillance, homeland security, and many more^1^. Following the first introduction of SERS as an analytical tool in 1984^2^, our laboratory has developed different types of plasmonics-active platforms^3–5^ for a wide variety of SERS applications in chemical sensing and biomedical applications^6, 7^. The SERS effect produces an enormous (~10^10^) enhancement of the Raman signal of the analyte molecules^8, 9^, which is attributed to the electromagnetic mechanism (EM) and the chemical mechanism (CM)^10, 11^. The EM enhancement exhibits the highest effect in the near vicinity of plasmonic noble metal nanostructures, where a strong local electric field generated on the surface of the plasmonic noble metal nanostructures by laser excitation, referred to as localized surface plasmon resonance (LSPR); the analyte molecule interaction with the strong electric field leads to an increase in the polarizability of the molecule, thereby significantly enhances the Raman signal ^12^. It has been well established that anisotropic nanoparticles generate intense localized electric fields at their sharp edge, tips, and intermetallic junction known as SERS “hot spot”. For this reason, a significant area of advancement has been devoted to the morphology control of the plasmonic noble metal nanoparticles to enhance the SERS signal^13^.

Among various sizes and shapes of nanoparticle systems, anisotropic gold nanostars (GNS) have attracted attention because of their sharp tips generating intense electric fields in comparison to other shapes of gold nanoparticles like gold nanospheres, nanorods, nanotriangles, and nanobipyramids^14–18^. GNS possesses a spherical core and tunable protruding branches, which lead to strong electromagnetic field enhancement at their branch tips. There are various GNS synthesis methods involving seedless or seed-mediated growth, surfactant-based or surfactant-free synthesis methods^19–21^. For instance, our laboratory first introduced the use of GNS as a SERS-enhancing platform^22^. However, the reported methods for GNS synthesis remain scarce for practical SERS application, which is mainly due to the polydispersity of the morphology and unclear design principle^23, 24^. Therefore, it is essential to fine-tune the GNS morphology to achieve highly monodispersed sharp-branched morphology for improving the application of SERS.

Another important aspect that needs to be addressed for the selection of SERS substrate is utilizing it for real-world on-site applications. Regarding the practical application of SERS on uneven irregular surfaces, flexible and transparent SERS substrates are promising compared to conventional hard glass or silicon SERS substrates^25, 26^. To date, there has been significant progress in the designing and fabrication of flexible SERS substrates, including polymer films, adhesive tapes, and thermoplastic materials as support materials for real-world applications such as pesticide detection on fruits and detection of the drug molecules on human skin^27–36^. However, most of the reported procedures are limited due to the long substrate preparation process and weak physical adsorption of the nanoparticles to the substrate, thus reducing the reproducibility of the SERS platform. To address these challenges, the focus of the present study is to develop a simple strategy to prepare a transparent, reproducible, flexible sensor patch. We believe that the combination of transparent flexible SERS substrate with precisely controlled GNS may provide an ultra-sensitive detection platform.

We presented a facile method to prepare a SERS patch based on multibranched sharp spiked GNS anchored onto a commercially available adhesive Scotch Tape. Herein, we have selected the surfactant-free GNS, which has greater flexibility for fine-tuning the shape, size, and spike morphology to improve the SERS sensitivity. It is widely believed that large-sized GNS with long and sharp spikes can generate multiple hotspots, which can enhance the electromagnetic field. To achieve a multibranched sharp spiked GNS morphology, we have utilized 20 nm seeds instead of traditionally used 12 nm seeds to generate large-sized GNS and fine-tuned the spike morphology by changing the concentration of AgNO_3_. Moreover, we employed FEM simulations to understand the effect of branch sharpness of GNS on electric field enhancements, which indicates that the electric field enhancement is increased with increasing the branch sharpness. From our experimental work, we have tuned the branch sharpness of GNS from 10 nm (GNS-1) to 2.5 nm (GNS-4). The SERS sensitivities for each type of GNS were evaluated using R6G as a model analyte, which exhibits that GNS-4 has the maximum SERS sensitivity. Finally, we demonstrated our SERS patch having the best SERS performance nanoparticle system (GNS-4) for potential real-life applications through an example showing SERS detection of CV on the fish scale. The results show the ultra-low detection of CV with the minimum detection limit as low as 1 pM.

## EXPERIMENTAL SECTION

### Materials and Characterization

Ascorbic acid, chloroauric acid (HAuCl_4_), silver nitrate (AgNO_3_, 99.8%) hydrochloric acid (HCl), trisodium citrate (Na_3_C_6_H_5_O_7_), CV, and R6G were purchased from Sigma-Aldrich. A fish was purchased from a local market. Milli-Q deionized (DI) water was used throughout the experiment. The STEM images of GNSs were acquired using Aberration Corrected STEM Thermo-Fisher Titan 80–300. UV-vis spectra were recorded using a Shimadzu UV-3600i spectrometer with 1-cm path length cuvettes at room temperature. TEM images were taken using the FEI Tecnai G^2^ Twin TEM system. SEM images were taken using FEI Verios 460 L.

### Synthesis of multibranched sharp spiked GNS

Multibranched spiky GNS was synthesized by following a modified version of a reported method^37^. Briefly, 20 nm gold seeds were first synthesized following a reported procedure^38^. For the growth of multibranched spiky GNS, 200 μL of 1 M HCl was added to a solution containing 50 mL of 1 mM HAuCl_4_ and 1 mL of 20 nm as-synthesized gold seeds solution. After that, we added a variable amount of AgNO_3_ solution at 3 mM concentration and 1 mL of 100 mM ascorbic acid to the solution. The solution was stirred for 2 minutes and used for further substrate preparation.

### Preparation of multibranched spiky GNS substrate

APTES-functionalized Si-wafer substrate was prepared according to the literature procedures^39^. The Si-wafer substrates were washed with Aqua Regia (HCl: HNO_3_ in a 3:1 ratio by volume) and rinsed with Milli-Q water three times. The Si-wafer substrates were further cleaned in ethanol with sonication three times and dried at 100 °C for 1 h in an oven. The cleaned Si-wafer substrates were then dipped in a 1% (v/v) ethanol solution of APTES in ethanol at 70 °C for 2 h. After that, the substrates were rinsed three times in ethanol with sonication to remove excess APTES and dried for 2 h at 100 °C in an oven. Then, the Si-wafer substrates were vertically immersed overnight into 50 mL GNS solution and washed with ultrapure water gently once and dried. After that, a commercially available adhesive tape was placed on the GNS substrate, pressed gently with the thumb, and removed at a 90° angle, resulting in successful transfer of the GNS from the Si-wafer to the adhesive substrate, generating the plasmonic SERS patch. The detection of analytes was performed by directly drop-casting onto the SERS patch, followed by evaporation of the solvent at room temperature.

### Raman Measurements

Raman measurements were performed by using a laboratory build portable Raman instrument having a 785-nm laser source (Rigaku Xantus TM-1 handheld Raman device), a fiber optic probe (InPhotonics RamanProbe), a spectrometer (Princeton Instruments Acton LS 785), and a CCD camera (Princeton Instruments PIXIS: 100BR_eXcelon). The laser power of the Rigaku Xantus TM-1 was set at 200 mW, and the exposure time was set at 1 sec.

### Finite Element Modeling

The wave optics module in COMSOL Multiphysics 6.0 was used to theoretically evaluate different nanoparticle morphologies. For this study, the optical properties of gold described by Johnson and Christy ^40^ were used for all nanoparticle domains. The optical properties of water described by Diamon and Masumura were used for all other regions ^41^. The simulation domain had a radius of 700nm. For each simulation, the nanoparticle model was excited by a linearly polarized plane wave propagating in the positive x direction. For all models, the core radius was 50 nm, and the length of all branches was 100nm. The normalized electric field for nanoparticle morphologies with a branch tip diameter ranging from 1nm to 8 nm in steps of 0.5 nm was simulated at 785 nm. The meshing of all domains was physics-solver controlled and set to extremely fine. All heat loss values were calculated by integrating the volume of the nanoparticle model with the resistive losses at the given wavelength.

## RESULTS and DISCUSSION

### Preparation of the SERS patches

In this study, we have used surfactant-free GNS for the preparation of SERS plasmonic patches because of their unique multibranched morphology and localized surface plasmon resonance (LSPR) properties for improving the SERS performance^17, 42, 43^. Indeed, the overall GNS morphology depends on various factors such as the concentrations of AgNO_3_, ascorbic acid, HAuCl_4_, and gold seeds. Among these parameters, seed size and AgNO_3_ play crucial roles in the evolution of the multi-branched sharp spikes^42^, where the large size seeds can generate multiple nucleation centers for the growth of spikes^43^, and AgNO_3_ plays an important role in making the spikes much sharper, which could be due to the underpotential deposition of Ag on the spikes reducing the diffusion of highly energetic gold atoms at the sharp tips toward the more energetically favorable spherical core of the GNS^42^. The SERS enhancement factor of GNS depends on several factors, such as the plasmon resonance peak maximum of the nanoparticles, and the number of hot spots^37, 44–47^. Therefore, it is imperative to fine-tune the GNS morphology to achieve maximum SERS enhancement. For instance, smaller-sized seeds can produce GNS with a plasmon resonance peak closer to the laser excitation wavelength (785 nm). However, smaller seeds can generate fewer nucleation centers, resulting in a smaller number of spikes, thereby reducing the overall impact of SERS. On the other hand, a larger seed size can produce more spikes. Nonetheless, due to its size, the plasmon resonance peak of the GNS becomes red-shifted to the NIR region, which may prevent us from taking advantage of the laser’s excitation wavelength^43^. To achieve maximum SERS enhancement, we, therefore, believe a moderate seed size would be optimum to generate a GNS morphology with a plasmon peak closest to the laser excitation wavelength (785 nm).

In this study, to prepare the large-sized multibranched sharp spiked GNS for improving the SERS performance of the flexible SERS patch, we have optimized the surfactant-free GNS synthesis by using 20 nm seeds. In addition, four different concentrations of AgNO_3_ (15, 30, 60, and 120 μM) were used to tune the GNS morphology and we achieved four different morphologies of GNSs (GNS-1, GNS-2, GNS-3, and GNS-4).

**Figure 2.**
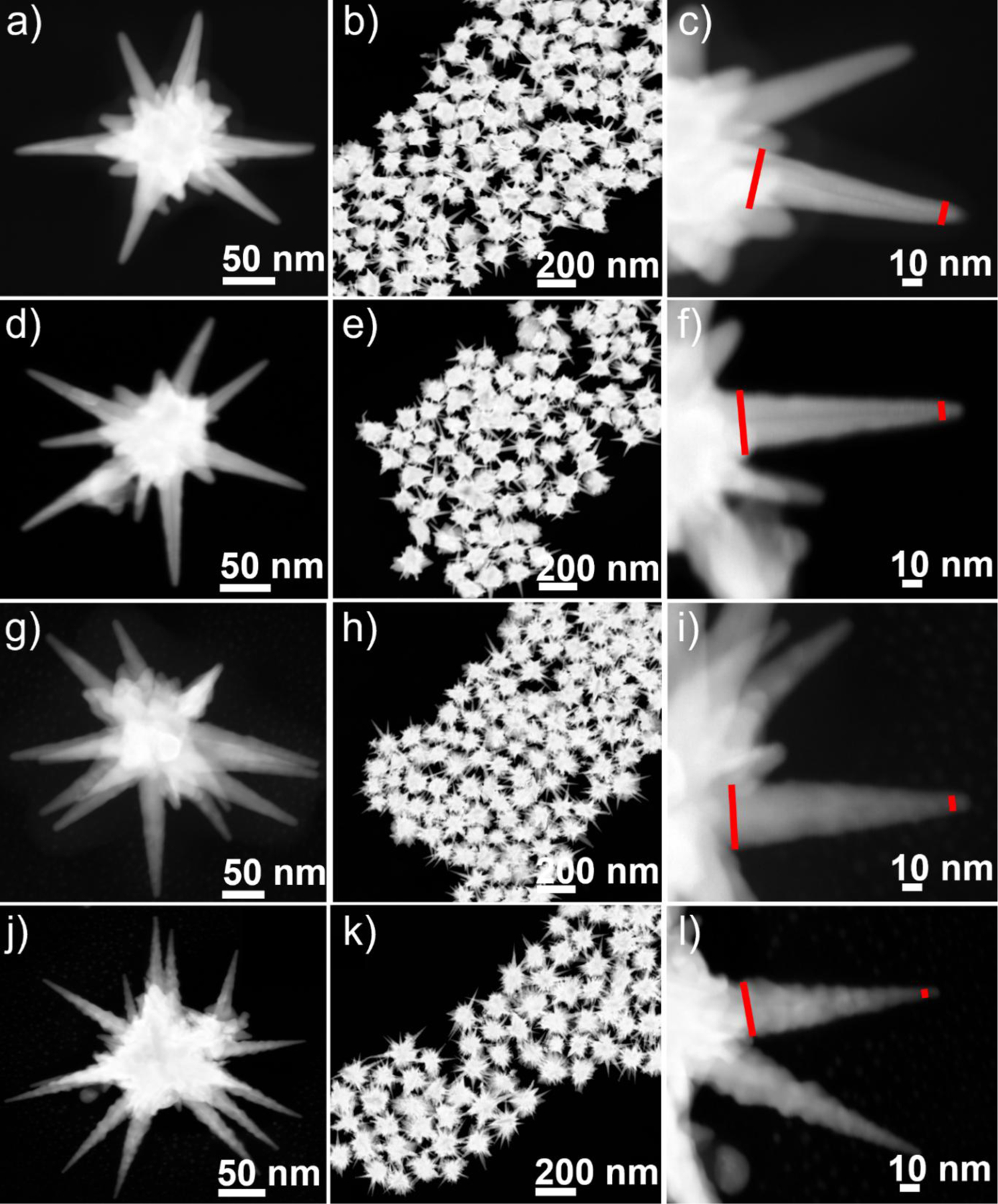
STEM images of GNS-1 (a-c), GNS-2 (d-f), GNS-3 (g-i), and GNS-4 (j-l) at different magnifications showing the different spiked morphology having average branch tip diameters 10, 7, 5, and 2.5 nm with high monodispersity of the GNSs.

**Figure 3.**
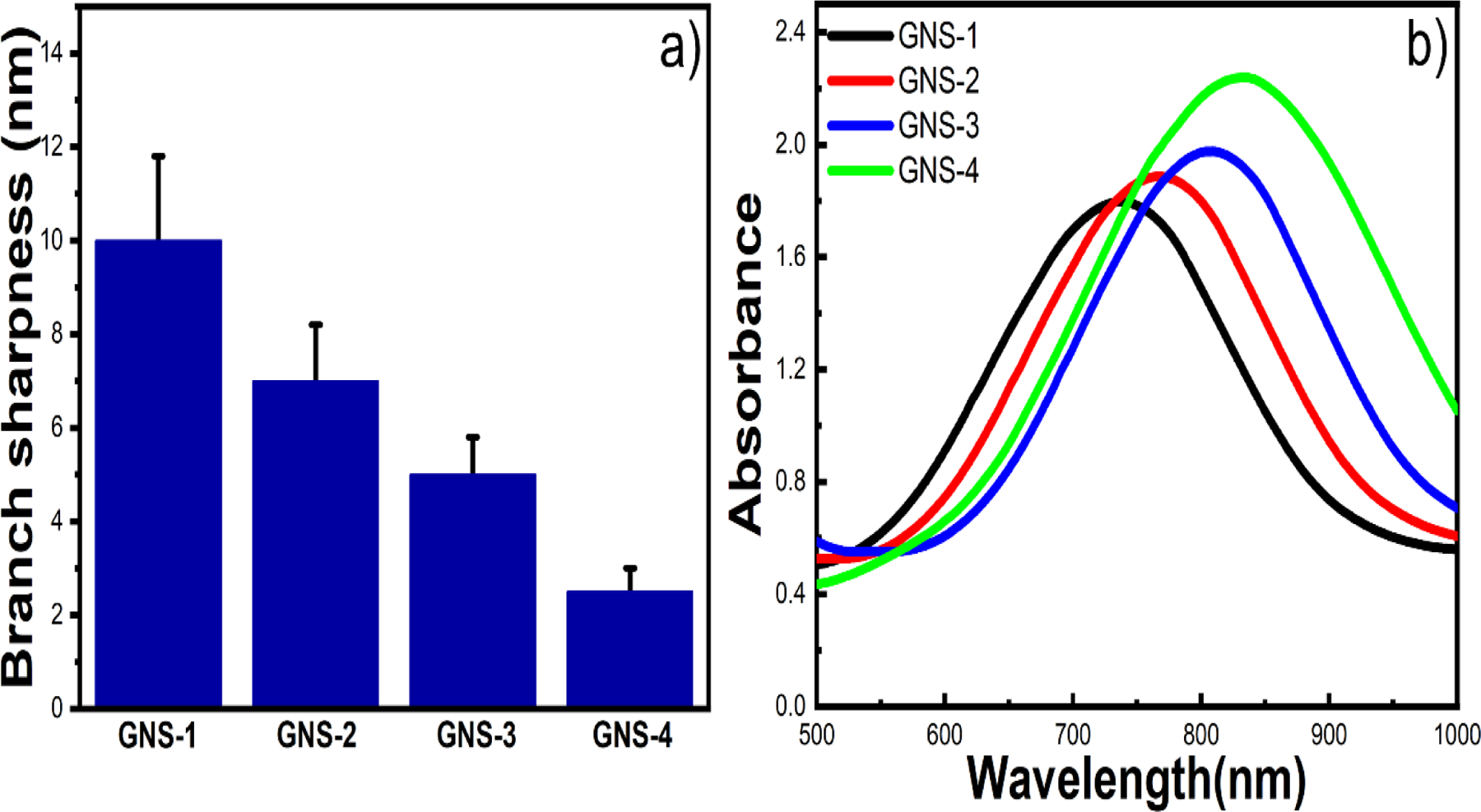
Branch sharpness of GNS-1, GNS-2, GNS-3, and GNS-4 (a). UV-Vis absorbance spectra of the GNSs indicating that the maximum plasmon peak position was red-shifted from GNS-1 to GNS-4 (b).

**Figure 4.**
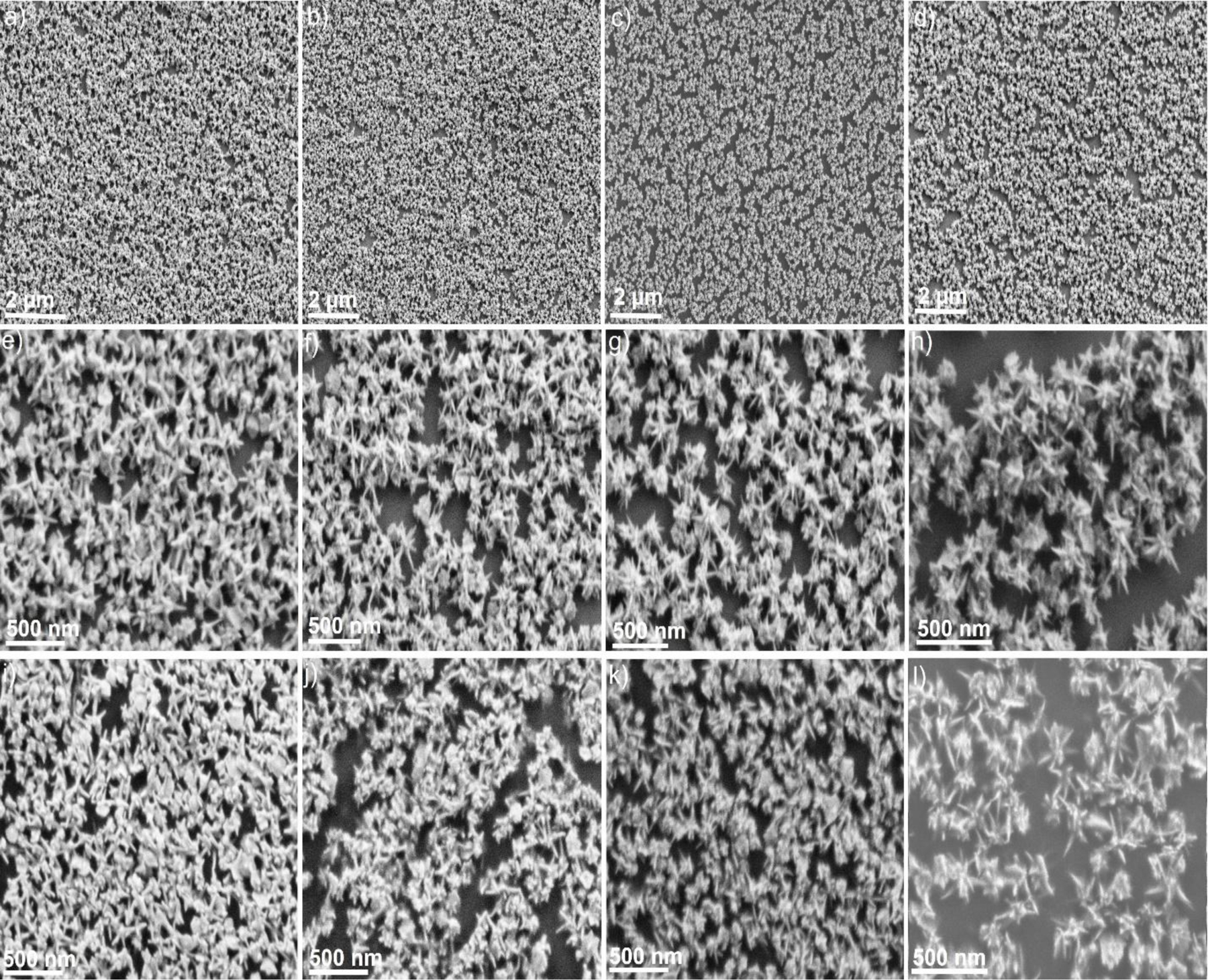
SEM images of the GNS-1 (a), GNS-2 (b), GNS-3 (c), and GNS-4 (d) showing high monodispersity of the GNSs distributed on the Si-wafer substrate(a-d). SEM images of the GNSs at high magnification showing the retained morphology of the GNSs (e-h). SEM images of the flexible SERS patches of the GNSs indicating that the morphology and dispersity were retained (i-l).

Figure S1 displayed the transmission electron microscopy (TEM) image of the gold nanospheres, where the average gold nanospheres size was measured up to 20 nm. We used the 20 nm gold nanospheres for multi-branched gold nanostars synthesis. It is reported that the branch sharpness can be tuned by changing the concentration of AgNO_3_^42^. To investigate the effect of AgNO_3_ on the large-size multibranched GNS, we have tuned concentrations of AgNO_3_. Figure 2 exhibited the STEM images of the GNSs (GNS-1, GNS-2, GNS-3, and GNS-4) at four different concentrations of AgNO_3_ (15, 30, 60, and 120 μM). Figures 2b, 2e, 2h, and 2k show the STEM images with multiple numbers of GNSs indicating that the synthesis of GNSs was highly monodisperse. The magnified STEM images (Figures 2c, 2f, 2i, and 2l) show that the width of the base of the spike was around 33 nm whereas the tip diameter was increased from GNS-1 to GNS-4. We measured the branch tip diameter and branch length of 100 different GNSs. The average branch tip diameter was determined to be 10±1.8, 7±1.4, 5±0.9, and 2.5±0.9 nm for GNS-1, GNS-2, GNS-3, and GNS-4, respectively (Figure 3a). The spike length of the GNSs were determined from the surface of the core to the tip of the GNSs. The average branch length was determined to be 95±14.8, 95±16.5, 98±12.6, and 103±15 nm for GNS-1, GNS-2, GNS-3, and GNS-4, respectively. It is evident from these measurements that the nanoparticles synthesized are uniform in size. The UV-Vis absorbance spectra revealed that the LSPR peak maximum of the GNS-1, GNS-2, GNS-3, and GNS-4 was red-shifted from 737 to 830 nm indicating that the morphology was changed (Figure 3b). Figure 4a–d displayed the scanning electron microscopy (SEM) images of the silicon water substrate with GNSs indicating that the GNSs were uniformly distributed creating ample hot spots to enhance the overall electric field. The magnified SEM images revealed that the morphology of the GNSs was retained (Figure 4e–h). More importantly, the SEM images of the flexible adhesive tape decorated with GNSs exhibited that the GNSs were successfully transferred onto the adhesive tape with retained morphology of the nanoparticles (Figure 4i–l). The SEM images of the GNS-4 flexible substrate at different magnifications show that the overall nanostar morphology has been preserved (Figure S2). In spite of this, we were unable to obtain very high-resolution SEM images of the GNS-4 tip due to the practical limitation of the SEM instrument. The detailed investigation to determine the tip morphology will be considered in further studies.

### Theoretical calculation of GNSs

**Figure 5.**
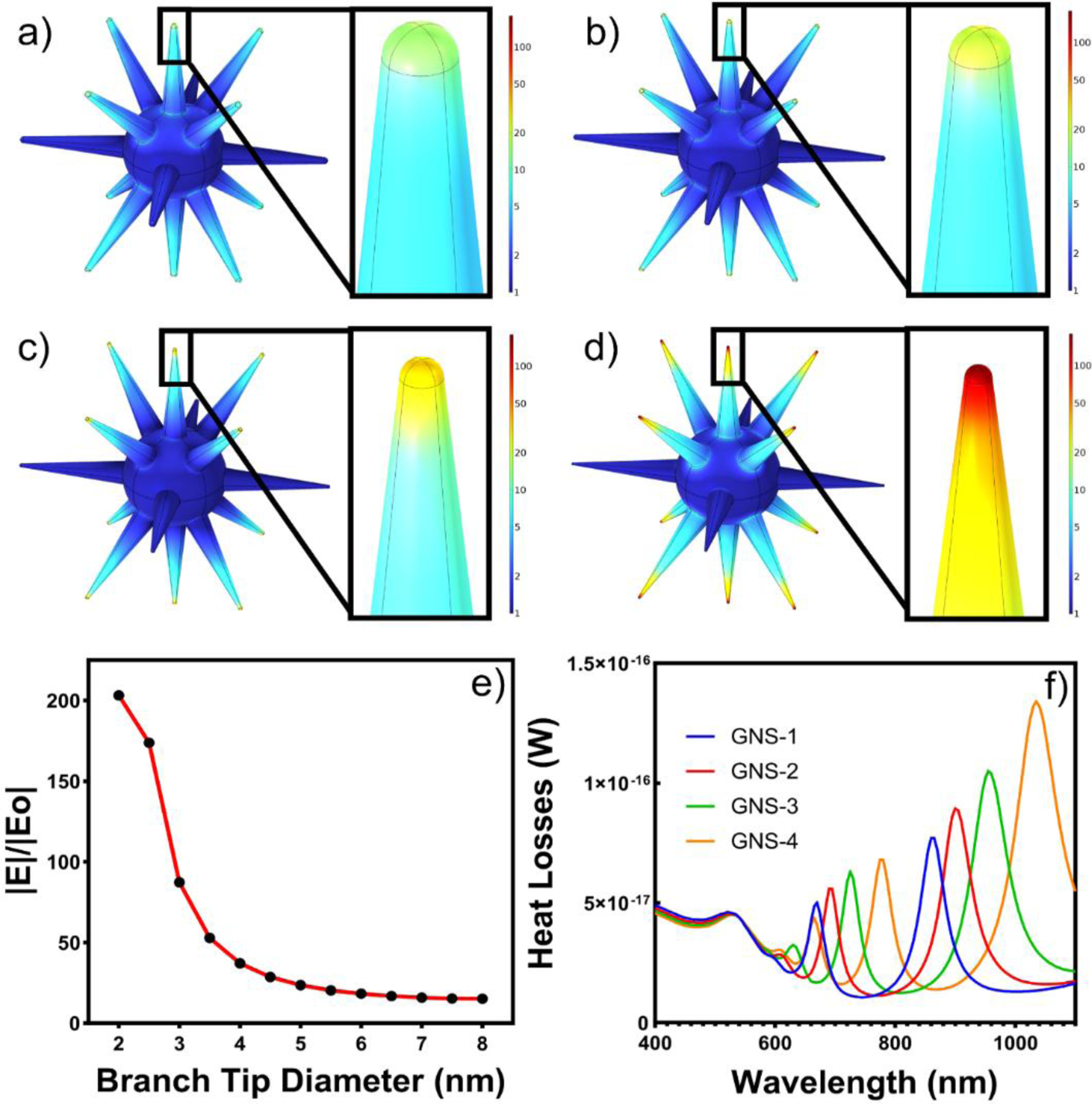
Normalized electric field at 785 nm for GNS-1 (a), GNS-2 (b), GNS-3 (c), and GNS-4 (d) with branch tip inset. Maximum normalized electric field generated by GNS model as a function of branch tip diameter (e). Heat loss spectra of models corresponding to GNS-1, GNS-2, GNS-3, and GNS-4 (f).

We have investigated the theoretical optical properties of different branch tip radii of the GNS morphologies by wave optics package in COMSOL Multiphysics 6.0 (Figure 5). GNS models in COMSOL were constructed with the same average branch tip diameter seen in each of the four GNS morphologies. These models are shown on the left of Figures 5a–d, with branch tip diameters of 2.5 nm, 4 nm, 5.5 nm, and 7nm. These models correspond to GNS-4, GNS-3, GNS-2, and GNS-1, respectively. The normalized electric field at 785 nm for each of the four GNS morphologies is shown on the right in Figures 5a–d, with an enlarged view of a representative branch tip in each inset. The diameter of the branch tip has an inverse relationship with the observed normalized electric field enhancement, denoted as |E|/|Eo|. Among the four representative models, GNS-4 generates the highest |E|/|Eo| value at 179.03 V/m. To further investigate the relationship between theoretical data and experimental results, we conducted simulations of GNS models with branch tip diameters ranging from 1 nm to 8 nm, in increments of 0.5 nm, at a wavelength of 785 nm. The findings of this parameter sweep are displayed in Figure 5e, where we observed the highest |E|/|Eo| value was generated by the GNS model with the smallest branch tip diameter. As the branch tip diameter increased, the maximum |E|/|Eo| values decreased, agreeing with the initial trend in the models corresponding to GNS-1, GNS-2, GNS-3, and GNS-4 morphologies. Although the maximum |E|/|Eo| value can offer guidance on the ideal nanoparticle morphology for SERS detection at a particular wavelength, it does not provide information for the entire particle. For this reason, we also computed the heat loss value for each model at 785 nm. In Figure 4f, we simulated the heat loss spectra for GNS-1, GNS-2, GNS-3, and GNS-4 models from 400 nm to 1100 and compared them to the experimental absorbance spectra shown in Figure 3b. Each GNS model had two prominent peaks in the heat loss spectra that redshifted as the branch tip diameter decreased. This same trend was observed in the experimental absorbance spectra for the corresponding GNS solutions. The relative peak broadness seen in the absorbance spectra compared to the simulated heat loss spectra is due to the heterogeneity of the experimental GNS synthesis. However, the strong agreement in both the location of the peaks and their spectral shift in response to altering the average branch tip diameter provided strong evidence that the simulated models accurately represented experimentally achievable GNS morphologies illustrated in Figure 2. These simulated results suggest that the experimentally observed GNS-4 morphology was the optimum GNS morphology which can generate the strongest SERS enhancement under 785 nm laser excitation. We have further investigated the effects of GNS branch length, where the local electric field was simulated for GNS-1, GNS-2, GNS-3, and GNS-4 models with branch lengths of 90 nm, 95 nm, 100 nm, 105 nm, and 110 nm between 700 nm and 800 nm incident light. The resulting heat losses spectra are shown in Figure S3a–f. As the branch length increases from 90 nm to 110 nm, the LSPR peak of all GNS morphologies in the spectral region of interest red-shifts. The branch length-averaged spectra for each base GNS morphology are shown in Figure S3f, where GNS-4 continues to have the greatest heat losses value, followed by GNS-1, GNS-3, and finally GNS-1.

### SERS performance of the flexible patches

**Figure 6.**
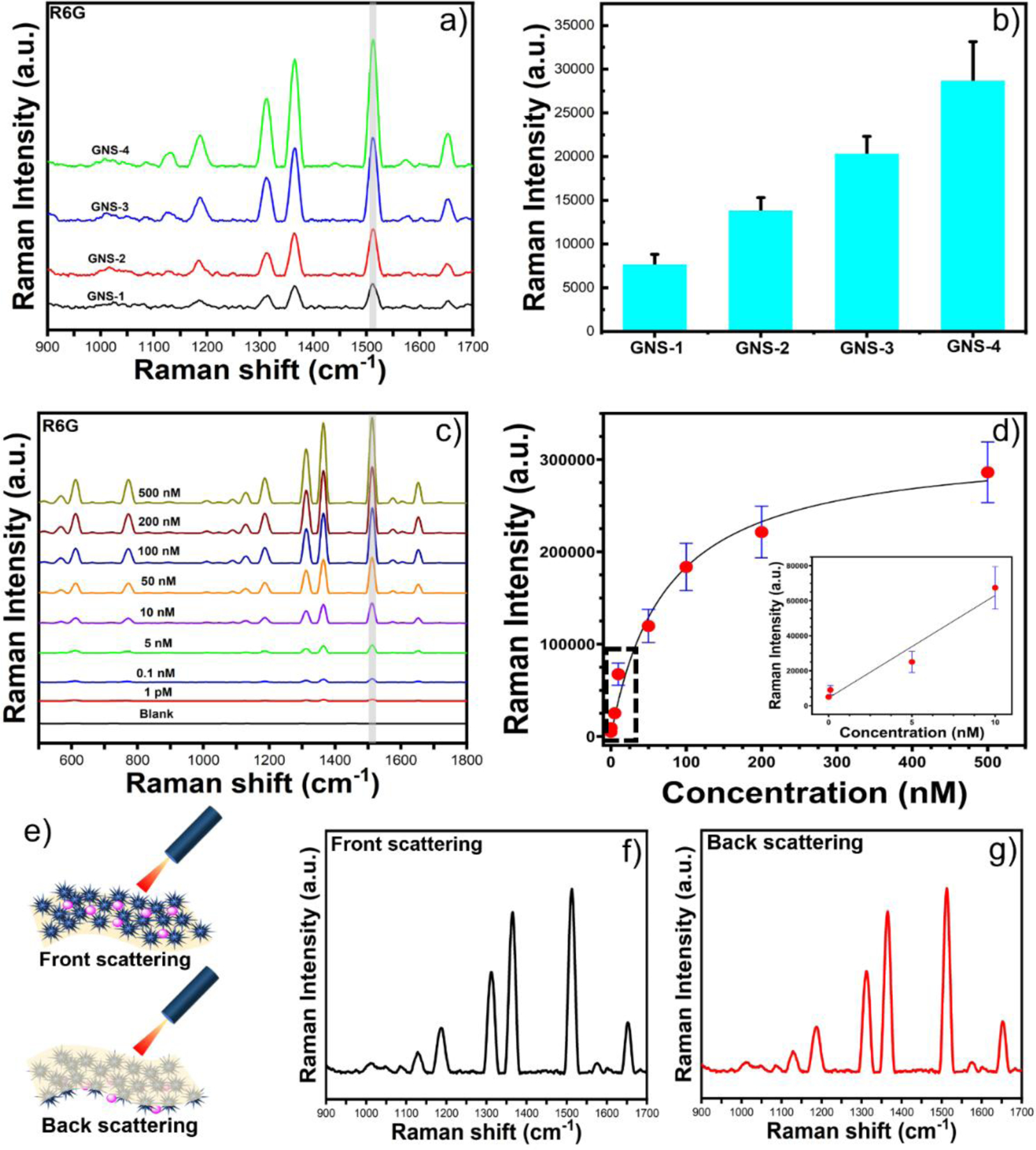
The SERS spectra of R6G at the concentration of 100 nM with different morphology of GNSs which show that GNS-4 has the maximum SERS enhancement (a). The SERS intensity of R6G at 1511 cm^−1^ for the GNS-1, GNS-2, GNS-3, and GNS-4 (b). The SERS spectra and the corresponding calibration curve of R6G at different concentrations ranging from 50 nM to 50 pM with the SERS patch having GNS-4 (c-d). Schematic of the front and back scattering (e), and the SERS spectra of R6G at the concentration of 100 nM for front and back scattering (f-g).

To validate the theoretical simulation work, we have experimentally investigated the SERS properties of different branch sharpness of GNSs at a 785 nm NIR excitation using a portable Raman instrument. Figure 6a shows the SERS peak intensity of R6G at 100 nM concentration with different GNSs. The most intense SERS peak of R6G at 1511 cm^−1^ was chosen to compare the SERS patches. As depicted in Figure 6b, GNS-4 shows a much stronger SERS enhancement than other GNSs. We also compared our GNS-4 flexible substrate with traditional 40 nm gold nanospheres (GNSP) flexible substrates. A similar procedure was followed for preparing GNSP flexible patches to that used for GNS-4. The SEM image of the GNSP flexible substrate is shown in Figure S4a. The SERS peak intensity at R6G at 1511 cm^−1^ was chosen to compare the SERS patches, which indicates that GNS-4 was almost seven times higher than the GNSP flexible substrate (Figure S4b). As expected, the GNS-4 substrate exhibited the maximum SERS enhancement, which might be probably due to the presence of sharp long spikes generating multiple spots and the LSPR maximum near the excitation wavelength at 785 nm.

Furthermore, we evaluated the analytical enhancement factor (AEF) for different branch-spiked GNSs Using the highest SERS signal of R6G at 1511 cm^−1^. The AEF of the GNSs was determined using the analytical enhancement factor formula, as shown in equation (1):

$$\text{AEF}=\left({\text{I}}_{\left(\text{SERS}\right)}/{\text{C}}_{\left(\text{SERS}\right)}\right)/\left({\text{T}}_{\text{Raman}}/{\text{C}}_{\text{Raman}}\right)$$

where I_(SERS)_ and I_Raman_ are the Raman peak intensities of the characteristic of R6G peak at 1511 cm^−1^ in the SERS spectrum and normal Raman spectrum, respectively. The C_(SERS)_ and C_Raman_ terms are the R6G concentration in the SERS spectrum and normal Raman spectrum, respectively. The enhancement factor from the GNS-1, GNS-2, GNS-3, and GNS-4 was calculated to be 1.2× 10^6^, 9.8× 10^7^, 1.4× 10^7^, and 6.2× 10^8^. We compared the enhancement factor of our spiky GNS substrate with the reported flexible SERS substrates (Table S1), which indicates that our SERS platform is more sensitive than the reported SERS substrates. We believe that our flexible chip comprising spiky gold nanostars generates intense electric fields in comparison to other different size and shape of gold or bimetallic gold-silver nanoparticles; thereby, achieving higher SERS enhancements.

Figure 6c shows the SERS spectra of R6G at different concentrations from 500 nM to 1 pM. The calibration curve (Figure 6d) between the concentration of R6G and the Raman intensity at 1511 cm^−1^ displayed a one-site specific binding equation as described below:

$$y=Bmax\times \frac{x}{Kd+x}$$

Where *y* is the SERS intensity of R6G, *Bmax* is the SERS intensity at the saturation coverage (Bmax= 317397), *Kd* is the equilibrium dissociation constant in the competitive adsorption process (*Kd* =73), and *x* is the concentration of R6G. The R^2^ value was 0.98. The inset image of the calibration curve shows a linear relationship between the SERS intensity versus the concentration of R6G in the concentration range from 10 nM to 1 pM. The LOD of R6G was calculated to 1 pM with a good signal-to-noise ratio (S/N= 3.5) indicating that ultra-high sensitivity was achieved for the flexible SERS substrate with GNS-4. To assess the reproducibility of the flexible SERS substrate, we investigated the SERS intensity of R6G of 30 different sample spots of a substrate. Figure S5 displays the SERS spectra of 100 nM R6G with the relative standard deviation (RSD) value calculated at 5.4 %, indicating that our SERS substrate possesses outstanding signal uniformity and excellent reproducibility.

For practical applications such as rapid detection of analytes on sample surfaces, the flexible substrate should possess excellent optical transparency such that the laser can easily pass through the transparent adhesive tape and then interact with plasmonic nanoparticles to generate an intense local electromagnetic field, which enhanced the Raman signal of analytes adsorbed on the plasmonic nanoparticle surface. To investigate the feasibility of the flexible SERS substrate with GNS-4 for *in situ* SERS analysis, front and back-side SERS measurements were performed with R6G at 100 nM concentration (Figure 6e). As shown in Figure 6f–g, the SERS spectra of the front and back sides of the SERS substrate were almost identical, indicating that good optical transparency was achieved by our SERS substrate, and it can be suitable for *in situ* SERS detection on irregular object surfaces. Overall, our flexible SERS substrate exhibits excellent SERS performance featuring high uniformity and excellent reproducibility, and possesses the capability of practical sensing application on substrates with arbitrary geometries.

### SERS detection of antifungal agent crystal violet (CV)

**Figure 7.**
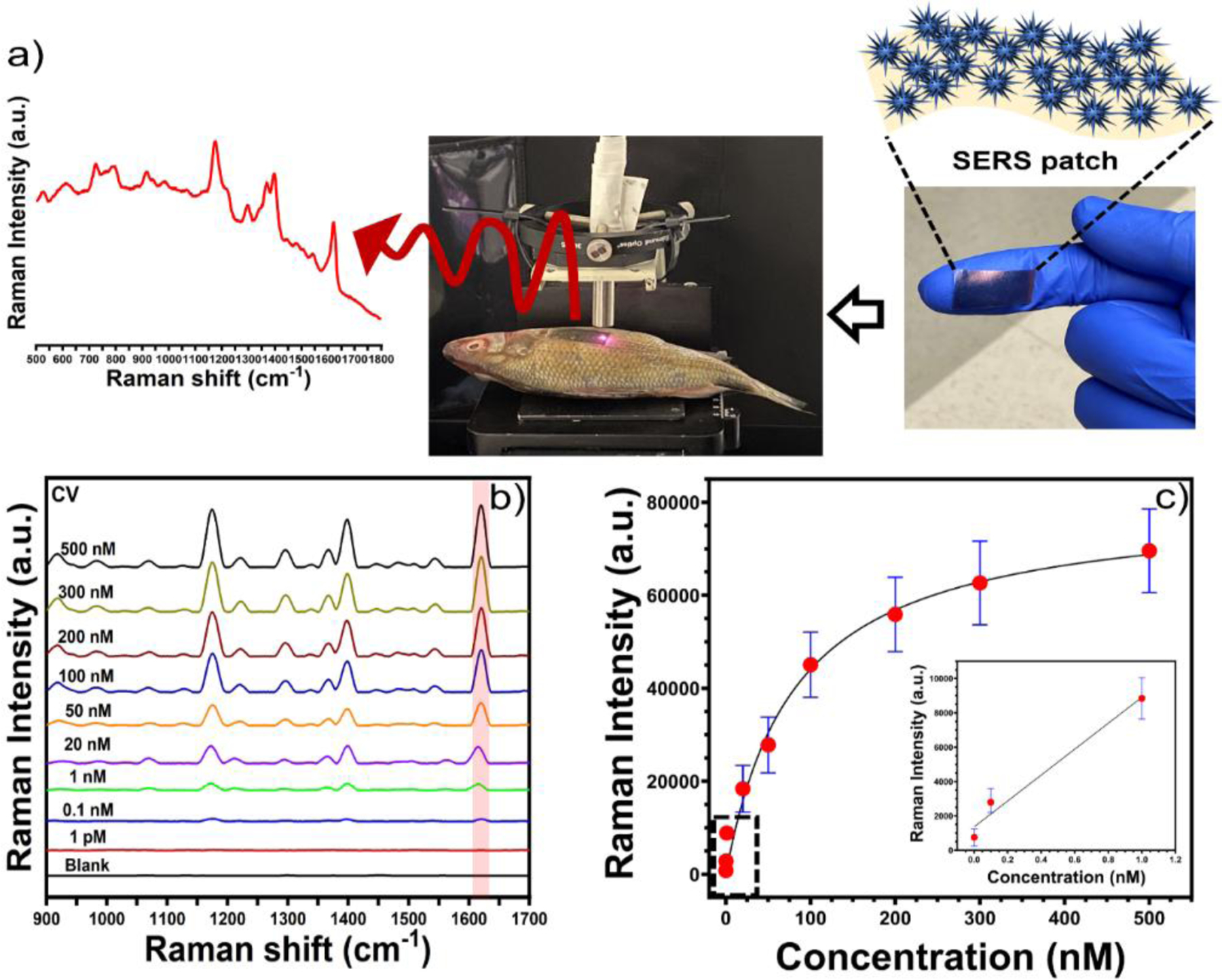
Schematic representation of the detection of CV on fish scales using SERS patch. The SERS spectra of CV at the concentration from 500 nM to 1 pM with GNS-4 flexible patch (a). The calibration curve of CV with GNS-4 flexible patch, which shows a linear relationship between SERS intensity at 1620 cm^−1^ at the concentration range from 1 nM to 1 pM of CV (b).

After determining the optimal morphology of GNS for SERS enhancement, we further investigated the quantitative SERS detection CV. CV is used as an effective antifungal agent in the aquaculture industry to prevent infections of bacteria and fungi in fish and fish eggs^48^. However, it could produce adverse effects on human health as they can generate carcinogenic compounds^49, 50^. Therefore, it is important to monitor the excessive usage of CV to protect human health, and water ecosystems. The most effective economical method would be to find CV directly on a fish scale. We have investigated our GNS-4 flexible patch substrate for the direct detection of CV on a fish scale (Figure 7a).

Figure 7b displays the SERS spectra of CV spiked in fish scale at different concentrations ranging from 500 nM to 1 pM. The most intense SERS peak of CV was located at 1620 cm^−1^, which is attributed to ring C-C stretching^51^. The Raman band at 1620 cm^−1^ was chosen as a calibration band to determine the calibration curve of CV (Figure 7c). The calibration curve showed a one-site-specific binding equation as described below:

$$y=Bmax\times \frac{x}{Kd+x}$$

Where *y* is the SERS intensity of CV, *Bmax* is the SERS intensity at the saturation coverage (Bmax= 79939), *Kd* is the equilibrium dissociation constant in the competitive adsorption process (*Kd* =81.41), and *x* is the concentration of CV. The inset image of the calibration curve shows a linear relationship between the SERS intensity versus the concentration of CV in the concentration range from 1 nM to 1 pM. The LOD of CV was calculated to 1 pM with a good signal-to-noise ratio (S/N= 3.5). These results illustrate that the flexible SERS substrate based on GNS-4 could be useful for the practical on site detection of CV on fish scale.

Furthermore, we conducted a control experiment examining the binding affinity of the GNS-4 to the flexible patch. In this experiment, we studied the SERS measurements twice on the fish scale using two different spots on the same flexible patch with CV at 500 nM concentration. Following one SERS measurement of CV at 500 nM concentration on a fish scale using one spot on the flexible patch, another SERS measurement of CV at 500 nM concentration was conducted at a different spot on the same flexible patch. Interestingly, we observed only around ~3 % reduction of the SERS enhancements, indicating that GNS-4 was firmly bonded to the flexible patch (Figure S6a). In addition, we studied the stability of the GNS-4 flexible substrate at room temperature for three weeks at seven-day intervals by measuring the SERS signal intensity of R6G at 1511 cm^−1^ at 500 nM. As depicted in Figure S6b, the SERS intensity of R6G at 1511 cm^−1^ at 500 nM was almost identical after three weeks of storage, indicating that the GNS-4 flexible substrate was stable for a long period. The results demonstrate that the GNS-4 flexible substrate exhibits excellent detection sensitivity, reproducibility, and stability.

We calculated the recovery (C2/C1×100%) of four different concentrations of CV detection on the fish scale by GNS-4 flexible patch, where C2 represents the spiked CV concentration and C1 represents the observed CV concentration^52^. As shown in Table S2, the recovery percentages of all samples ranged from 89 to 114 % with a relative standard deviation (RSD) of less than 10 % indicating that our flexible chip can be applied to practical SERS detection of CV in aquatic products.

We have further investigated another analyte malachite green (MG) commonly used as an antiparasitic drug in the fishery industry^53^. Figure S7a displays the SERS spectra of MG spiked in fish scale at different concentrations ranging from 500 nM to 0.1 nM. We have selected the peak at 1172 cm^−1^, which is attributed to in-plane vibrations of the ring C-H vibration mode^54^, for the determination of calibration curve (Figure S7b). The inset image of the calibration curve shows a linear relationship between the SERS intensity versus the concentration of MG in the concentration range from 10 nM to 0.1 nM. The LOD of MG was calculated to 0.1 nM with a good signal-to-noise ratio (S/N= 3.5). It is reported that the CV and MG concentrations in real catfish and trout tissue samples were in the range of 20–30 nM^55, 56^. Our flexible SERS substrate’s LOD values for CV and MG were 1 pM and 0.1 nM, which is much lower than the real fish samples, indicating that our detection method is suitable for rapid sensitive CV and MG detection. It is worth noting that these results indicate that the flexible SERS substrate based on GNS-4 could be useful in multiplexing the detection of analytes on uneven curved surfaces. Based on these results, the flexible SERS patch shows improved reproducibility and sensitivity over previously reported work, indicating that the SERS patch may be applied to complex surfaces for sensitive detection of target molecules^57–59^.

## CONCLUSION

In summary, we demonstrated an easy, low-cost method to fabricate a standard office-grade adhesive tape with highly sensitive SERS nanoparticle: multibranched sharp spiked gold nanostars to achieve a transparent, flexible, and ultra-sensitive SERS substrate. The optimal SERS sensitivity of the different branch sharpness of GNS was investigated theoretically and experimentally, which shows that the sharp spiked morphology: GNS-4 exhibited the highest SERS signal intensity. Due to the presence of multiple hot spots in the GNS-4, the as-prepared SERS flexible substrate exhibited high EF (6.2× 10^8^) with excellent reproducibility having RSD of 5 % for the detection of an analyte R6G. Moreover, our SERS platform shows good reproducibility with a RSD of SERS intensity of about 5.4 % for 30 spot measurements. As proof of concept, we have applied our flexible SERS substrate for detection of an antifungal agent CV directly on the fish surface, which shows an ultralow detection limit of CV up to 1 pM. Overall, our finding opens a new avenue for rapid, point-of-care, cost-effective, and ultra-sensitive SERS detection of analytes in the field of food safety, environmental monitoring, and national security.

## Supplementary Material

SI

## Figures and Tables

**Figure 1.**
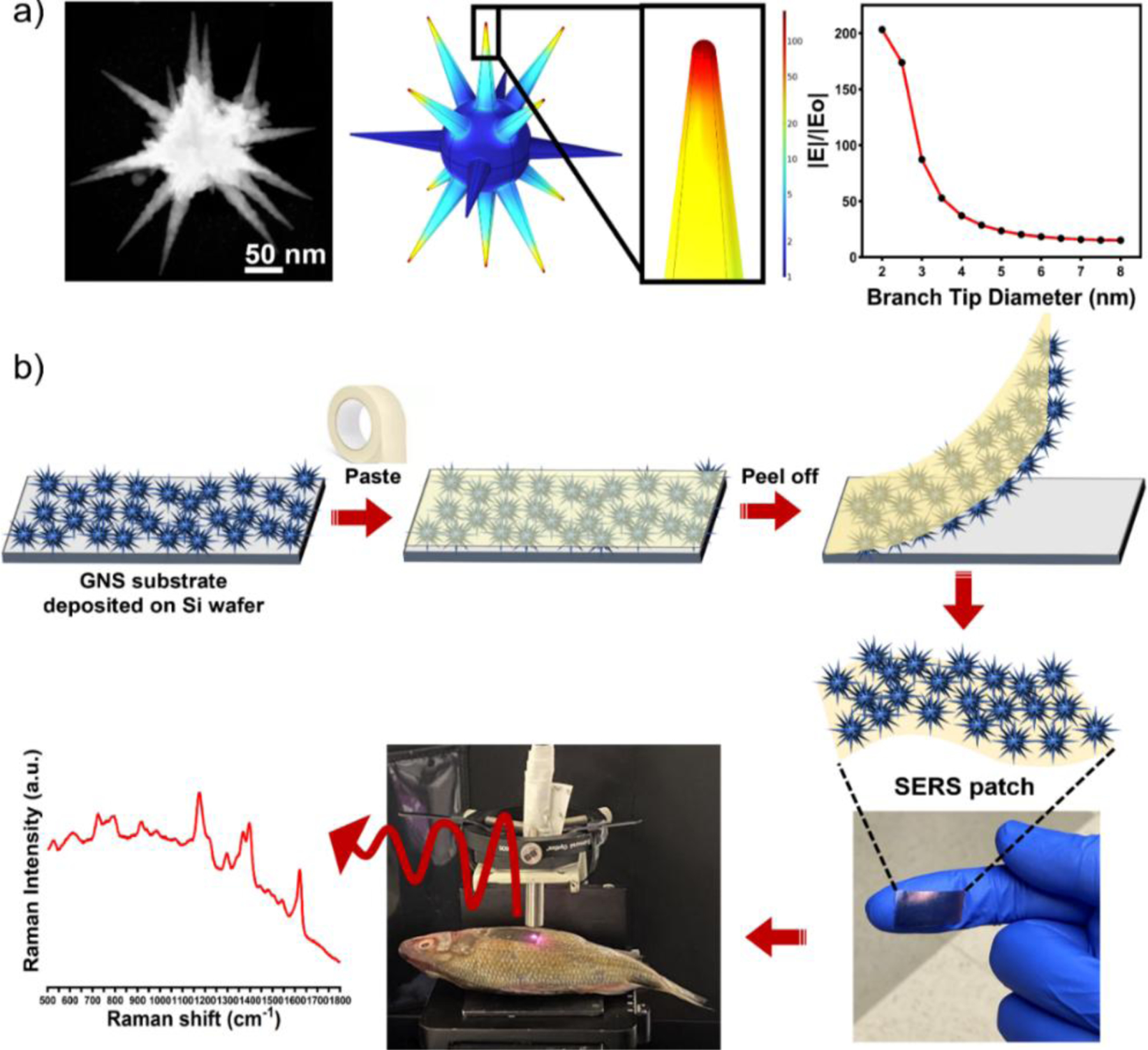
STEM images of GNS4, the normalized electric field for GNS-4 at 785 nm, and electric field enhancement (a). Schematic of the SERS patch preparation and direct SERS measurement of CV on fish scales (b).
