# Supplementary material for: A Simple Low-Cost Flexible Plasmonic Patch Based on Spiky Gold Nanostars for Ultra-Sensitive SERS Sensing
Source: Analyst. Author manuscript; Available in PMC 2026 Jan 6. (PMC12768891; doi:10.1039/d3an02246c)
Supplement: SI [file NIHMS1973631-supplement-SI.docx]

**Supporting Information**

**A Simple Low-Cost Flexible Plasmonic Patch Based on Spiky Gold Nanostars for Ultra-Sensitive SERS Sensing**

*Supriya Atta, ^a, b^ Aidan J. Canning, ^a, b^ Tuan Vo-Dinh ^a,^ ^b, c‡^*

^a^ Fitzpatrick Institute for Photonics, ^b^ Department of Biomedical Engineering, ^c^ Department of Chemistry, Duke University, Durham, NC 27708, USA.

‡ Correspondence author: tuan.vodinh@duke.edu


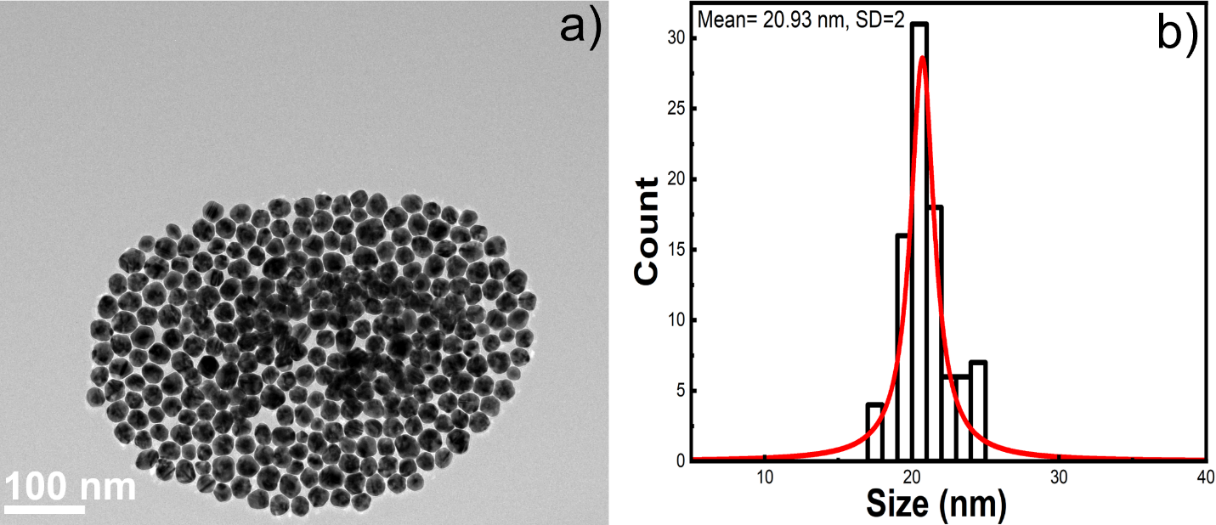


**Figure S1.** TEM image of gold seeds (a) and size distribution of them (b).


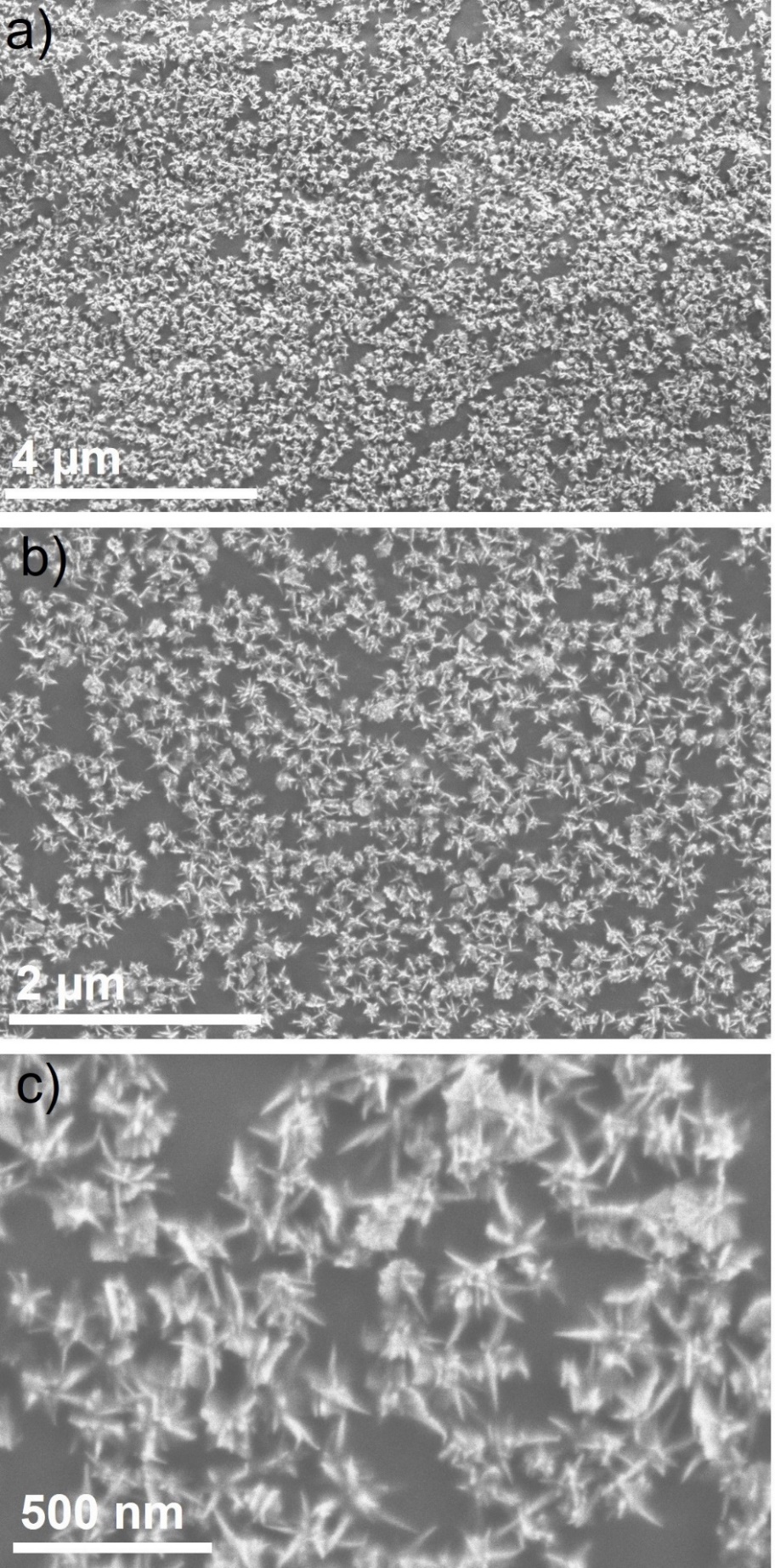


**Figure S2.** SEM image of the flexible substrate containing GNS- 4 at different magnification (a-c).


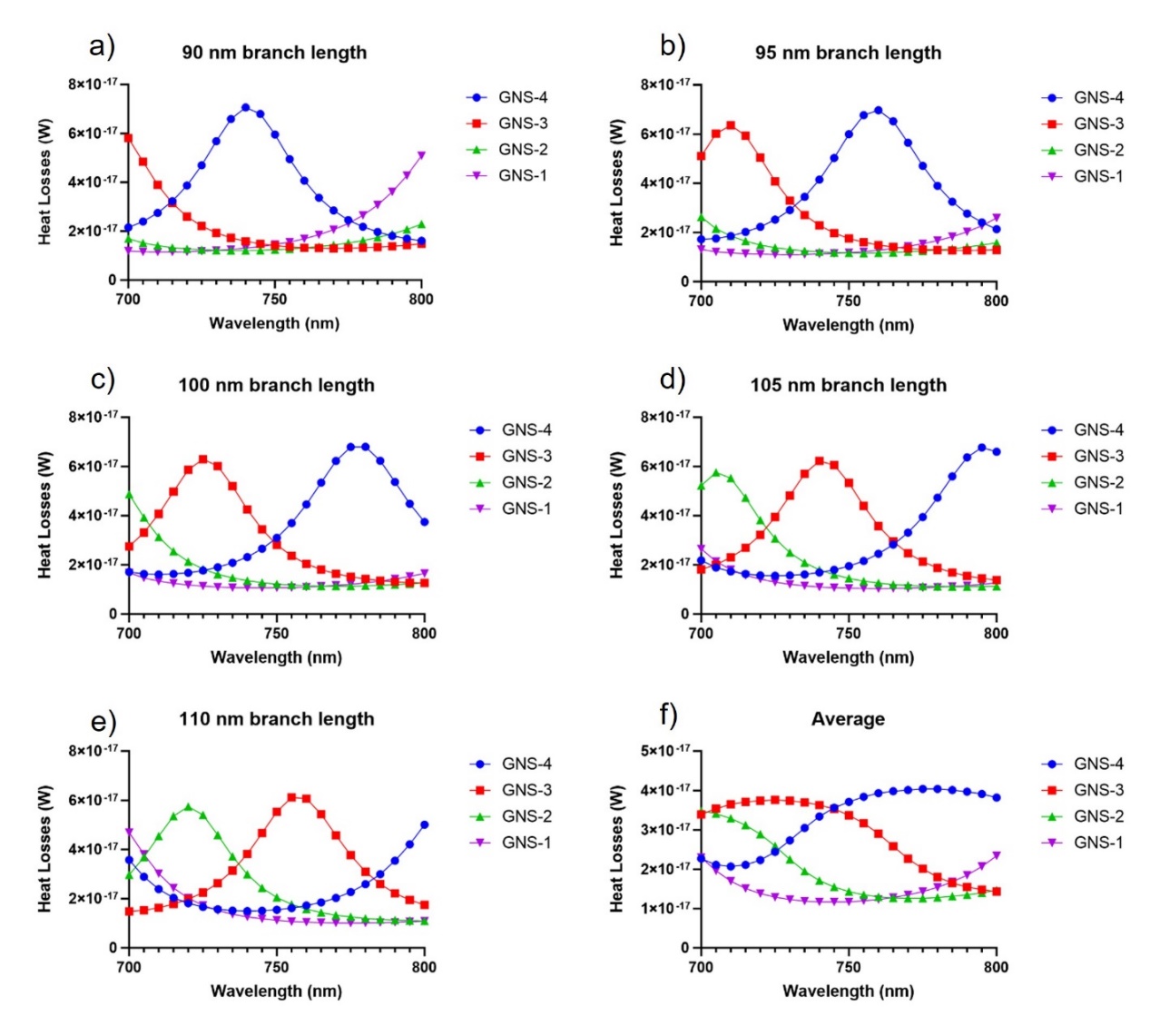


**Figure S3**. Heat losses spectra of different GNS morphologies with varying branch lengths. a) 90 nm branch length. b) 95 nm branch length. c) 100 nm branch length. d) 105 nm branch length. e) 110 nm branch length. f) Average of all branch lengths.


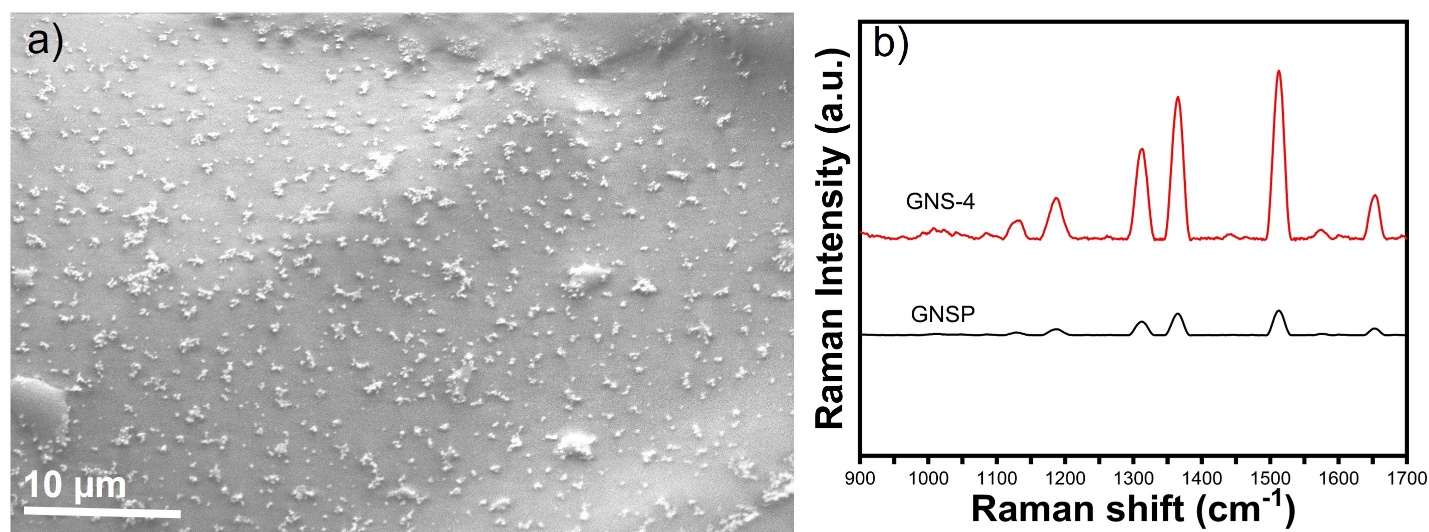


**Figure S4.** SEM image of the flexible substrate containing gold nanospheres (a). SERS spectra of R6G with GNSP and GNS-4 flexible substrate (b).


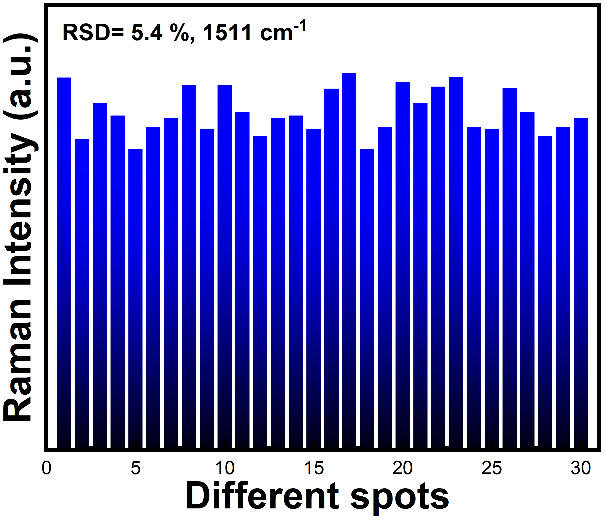


**Figure S5.** The reproducibility of SERS signals of R6G with GNS-4.


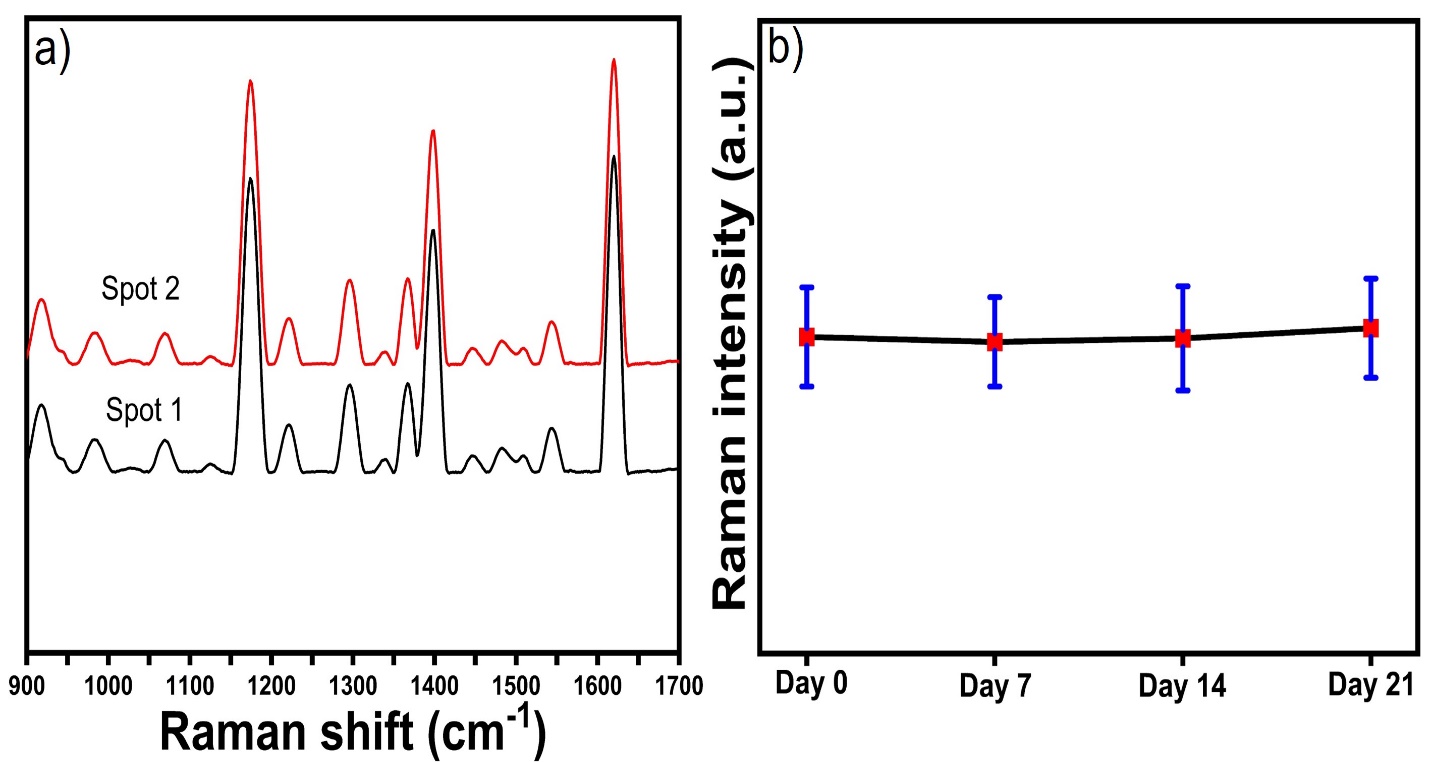


**Figure S6.** SERS spectra of CV at 500 nM concentration of two different spots on the same flexible patch (a). Stability of the GNS-4 flexible SERS substrate (b).


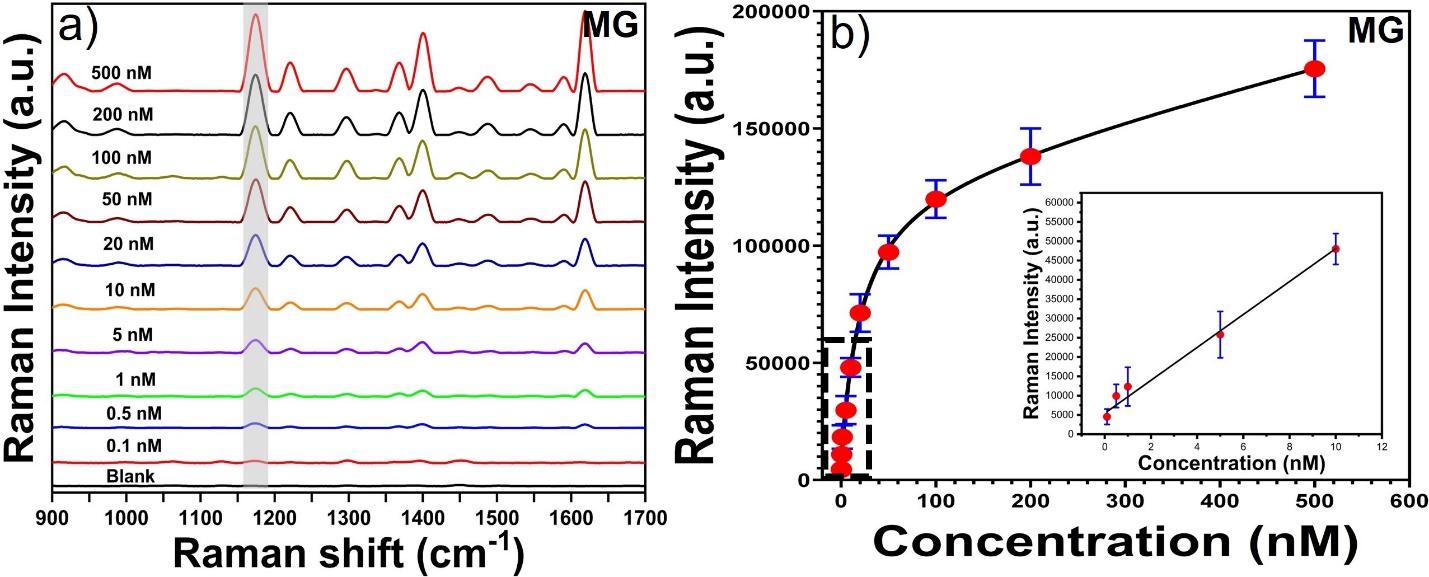
**Figure S7.** The SERS spectra of MG at the concentration from 500 nM to 0.1 nM with GNS-4 (a). The calibration curve of MG with GNS-4, which shows a linear relationship between SERS intensity at 1178 cm^-1^ at the concentration range from 0.1 nM to 10 nM of MG (b).

**Table S1.** Literature survey for enhancement factor determination of different flexible SERS substrate.

| Flexible SERS substrate | Enhancement factor (EF) | Reference |
| --- | --- | --- |
| Gold nanoparticles (Au NPs) | 1.3 × 10^5^ | ^1^ |
| Silver nanoparticles (Ag NPs) | ~1.0 × 10^5^ | ^2^ |
| Silver nanoparticles integrated with microlens | 10^7^ | ^3^ |
| Bimetallic Au@Ag plasmonic chip | 3.14 × 10^6^ | ^4^ |
| AuNPs@ polyimide heating chips | 5.5×10^5^ | ^5^ |
| Nanostructure array with 30 nm gold coating | 1.21 × 10^7^ | ^6^ |
| Silver-nanoparticle-grafted wrinkled polydimethylsiloxane (AgNPs@W-PDMS) | 6.11 × 10^6^ | ^7^ |
| Gold triangular nanoprisms (Au TNPs) | 4.5 ×10^7^ | ^8^ |
| Silver and gold core-shell nanoparticles | 1.07 × 10^7^ | ^9^ |
| Gold nanostar arrays | 1.9 × 10^8^ | ^10^ |
| Ag Nanocubes | 3.43 × 10^6^ | ^11^ |
| Spiky gold nanostars | **6.2 ×10^8^** | **This work** |

**Table S2.** Recovery percentage of the SERS measurement of CV on fish scale. *

| Spiked Concentration (nM) | Observed Concentration  (nM) | Recovery (%) | RSD (%) |
| --- | --- | --- | --- |
| 100 | 109.3 | 109.3 | 8.8 |
| 10 | 9.95 | 99.5 | 5.5 |
| 1 | 0.89 | 89 | 6.4 |
| 0.01 | 0.0114 | 114 | 5.8 |

* The measurements were repeated three times each.

**References**

1. Chen, J.; Huang, Y.; Kannan, P.; Zhang, L.; Lin, Z.; Zhang, J.; Chen, T.; Guo, L., Flexible and Adhesive Surface Enhance Raman Scattering Active Tape for Rapid Detection of Pesticide Residues in Fruits and Vegetables. *Analytical Chemistry* **2016,** *88* (4), 2149-2155.

2. Korkmaz, A.; Kenton, M.; Aksin, G.; Kahraman, M.; Wachsmann-Hogiu, S., Inexpensive and Flexible SERS Substrates on Adhesive Tape Based on Biosilica Plasmonic Nanocomposites. *ACS Applied Nano Materials* **2018,** *1* (9), 5316-5326.

3. Yang, F.; Wen, P.; Tang, L.; Wang, R.; Wang, Y.; Li, D.; Xu, Y.; Chen, L., A flexible surface-enhanced Raman Spectroscopy chip integrated with microlens. *Spectrochimica Acta Part A: Molecular and Biomolecular Spectroscopy* **2023,** *287*, 122129.

4. Wang, K.; Sun, D.-W.; Pu, H.; Wei, Q.; Huang, L., Stable, Flexible, and High-Performance SERS Chip Enabled by a Ternary Film-Packaged Plasmonic Nanoparticle Array. *ACS Applied Materials & Interfaces* **2019,** *11* (32), 29177-29186.

5. Wang, Z.; Dai, Y.; Zhou, X.; Liu, Z.; Liu, W.; Huang, L.; Yuan, M.; Cui, S.; He, X., Fabrication of flexible AuNPs@ polyimide heating chips for in situ explosives SERS sensing in nature samples. *Talanta* **2023,** *258*, 124460.

6. Zhang, C.; Yi, P.; Peng, L.; Lai, X.; Chen, J.; Huang, M.; Ni, J., Continuous fabrication of nanostructure arrays for flexible surface enhanced Raman scattering substrate. *Scientific Reports* **2017,** *7* (1), 39814.

7. Zhang, H.; Zhao, N.; Li, H.; Wang, M.; Hao, X.; Sun, M.; Li, X.; Yang, Z.; Yu, H.; Tian, C.; Wang, C., 3D Flexible SERS Substrates Integrated with a Portable Raman Analyzer and Wireless Communication for Point-of-Care Application. *ACS Applied Materials & Interfaces* **2022,** *14* (45), 51253-51264.

8. Masterson, A. N.; Hati, S.; Ren, G.; Liyanage, T.; Manicke, N. E.; Goodpaster, J. V.; Sardar, R., Enhancing Nonfouling and Sensitivity of Surface-Enhanced Raman Scattering Substrates for Potent Drug Analysis in Blood Plasma via Fabrication of a Flexible Plasmonic Patch. *Analytical Chemistry* **2021,** *93* (4), 2578-2588.

9. Liu, X.; Ma, J.; Jiang, P.; Shen, J.; Wang, R.; Wang, Y.; Tu, G., Large-Scale Flexible Surface-Enhanced Raman Scattering (SERS) Sensors with High Stability and Signal Homogeneity. *ACS Applied Materials & Interfaces* **2020,** *12* (40), 45332-45341.

10. Park, S.; Lee, J.; Ko, H., Transparent and Flexible Surface-Enhanced Raman Scattering (SERS) Sensors Based on Gold Nanostar Arrays Embedded in Silicon Rubber Film. *ACS Applied Materials & Interfaces* **2017,** *9* (50), 44088-44095.

11. Li, L.; Chin, W. S., Rapid Fabrication of a Flexible and Transparent Ag Nanocubes@PDMS Film as a SERS Substrate with High Performance. *ACS Applied Materials & Interfaces* **2020,** *12* (33), 37538-37548.
